# ‘Image-navigated 3-dimensional late gadolinium enhancement cardiovascular magnetic resonance imaging: feasibility and initial clinical results’

**DOI:** 10.1186/s12968-017-0418-7

**Published:** 2017-12-04

**Authors:** Konstantinos Bratis, Markus Henningsson, Chrysanthos Grigoratos, Matteo Dell’Omodarme, Konstantinos Chasapides, Rene Botnar, Eike Nagel

**Affiliations:** 1 Division of Imaging Sciences and Biomedical Engineering, King’s College London, London, UK; 2 Fondazione G. Monasterio CNR-Regione Toscana, Pisa, Italy; 3 Department of Physics, University of Pisa, Pisa, Italy; 4 Circle Cardiovascular Imaging, Calgary, Canada; 5 Institute for Experimental and Translational Cardiovascular Imaging, Frankfurt/Main, Germany

**Keywords:** 3D late gadolinium enhancement, Image-navigated, Cardiovascular  magnetic resonance

## Abstract

**Background:**

Image-navigated 3-dimensional late gadolinium enhancement (iNAV-3D LGE) is an advanced imaging technique that allows for direct respiratory motion correction of the heart. Its feasibility in a routine clinical setting has not been validated.

**Methods:**

Twenty-three consecutive patients referred for cardiovascular magnetic resonance (CMR) examination including late gadolinium enhancement (LGE) imaging were prospectively enrolled. Image-navigated free-breathing 3-dimensional (3D) T1-weighted gradient-echo LGE and two-dimensional (2D LGE) images were acquired in random order on a 1.5 T CMR system. Images were assessed for global, segmental LGE detection and transmural extent. Objective image quality including signal-to-noise (SNR), contrast-to-noise (CNR) and myocardial/blood sharpness were performed.

**Results:**

Interpretable images were obtained in all 2D–LGE and in 22/23 iNAV-3D LGE exams, resulting in a total of 22 datasets and 352 segments. LGE was detected in 5 patients with ischemic pattern, in 7 with non-ischemic pattern, while it was absent in 10 cases. There was an excellent agreement between 2D and 3D data sets with regard to global, segmental LGE detection and transmurality. Blood-myocardium sharpness measurements were also comparable between the two techniques. SNR_blood_ and CNR_blood-myo_ was significantly higher for 2D LGE (*P* < 0.001, respectively), while SNR_myo_ was not statistically significant between 2D LGE and iNAV-3D LGE.

**Conclusion:**

Diagnostic performance of iNAV-3D LGE was comparable to 2D LGE in a prospective clinical setting. SNR_blood_ and CNR_blood-myo_ was significantly lower in the iNAV-3D LGE group.

## Background

Cardiovascular magnetic resonance (CMR) late gadolinium enhancement imaging (LGE) is the standard of reference for the detection of myocardial necrosis [1]. The principle of LGE imaging is based on increased signal intensity in the damaged myocardium (compared to healthy) due to the increased extracellular space in necrotic tissue. The development of inversion recovery techniques together with electrocardiographic (ECG)-synchronization and breath holding has dramatically improved contrast between infarcted and normal myocardium [2].

Historically, a two-dimensional (2D) inversion recovery fast spoiled gradient-echo sequence has been applied for LGE imaging, necessitating multiple breath holds with potential slice misregistration and constraints on spatial resolution and signal-to-noise ratio (SNR) [3]. More recently, the development of 3-dimensional (3D) imaging has enabled data acquisition for the entire heart in a single scan [4–6]. Previously proposed 3D LGE methods included accelerated and extended breath-hold scans with similar constraints on spatial resolution and SNR as 2D LGE [7, 8]. This may be particularly detrimental to the detection of subendocardial scar which requires high resolution LGE. Alternatively, 3D LGE can be acquired during free-breathing which permits high resolution 3D LGE of the whole heart. To reduce respiratory induced motion artifacts, compensation techniques can be employed including the use of respiratory bellows signal [9] or diaphragmatic navigator [10–14]. Nevertheless, these techniques have limited motion estimation accuracy as they only indirectly measure the respiratory motion of the heart.

In order to address the limitations of the diaphragmatic navigator, respiratory self-navigation has been developed. With this strategy, the position of the heart itself is monitored over time. Initially developed for coronary artery visualization [15], this technique has been used for assessment of complex congenital cardiac malformations [16, 17]. More recently, image-navigation has been proposed for respiratory motion compensation in CMR, which provides direct respiratory motion tracking of the heart and can be combined with respiratory gating algorithms to improve robustness to motion [18].

The aim of this study was to prospectively examine the feasibility of image-navigated 3D LGE CMR (iNAV-3D LGE) for the detection of LGE in a routine clinical setting and perform a head-to-head comparison against conventional 2D LGE.

## Methods

### Patients

Consecutive patients with known or suspected heart disease that underwent a CMR examination including LGE imaging at our facility were enrolled from February 2014 to February 2015. Patients were required to be ≥18 years of age and have no contraindications to gadolinium contrast, inclusive of an estimated glomerular filtration rate ≤ 60 ml/min/1.73 m^2^. Patients with atrial fibrillation were excluded. Written informed consent was obtained from all participants. The institutional ethics board at Guy’s & St Thomas’ Hospital, King’s College London approved the study.

### CMR protocol

All participants were examined in supine position in a 1.5 Tesla CMR system (Gyroscan Intera, Philips Medical Systems, Best, The Netherlands) using a 32-element phased array cardiac synergy coil. The imaging protocol included cine imaging, standard 2D LGE imaging and iNAV-3D LGE imaging. 2D and iNAV-3D LGE image acquisitions were performed in random order to cancel out differences due to acquisition time after contrast agent injection.

**Fig. 1:**
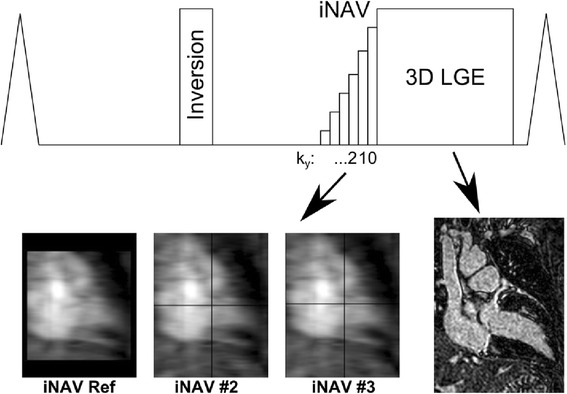
Pulse sequence diagram of image-navigated (iNAV)-3D late gadolinium enhancement (LGE) acquisition. The iNAV-3D was acquired during the ramp-up pulses of the LGE sequence, using a linear flip angle ramp and a high-low profile order. This means the higher k_y_ lines were acquired first and k_y_ = 0 acquired last to ensure that the centre line of k-space was acquired with the maximum flip angle and close temporal proximity to the 3D LGE acquisition. The first iNAV acquisition was used as a reference (iNAV Ref), and the local shim geometry used to automatically select the tracked region of interest which was registered to every subsequent iNAV (#2-#3) using normalized cross correlation. The registration results were displayed in real-time by overlaying crosshairs corresponding to the calculated motion onto the iNAVs. The registration provided translational motion estimation in foot-head and left-right direction for each 3D LGE k-space segment and motion correction was applied to the raw data as a linear phase shift. iNAV-3D LGE: Image-Navigated Late Gadolinium Enhancement

Cine images were acquired using a ECG-gated balanced steady-state free precession based pulse sequence in serial short-axis slices from the atrioventricular annulus to the apex at 10 mm intervals, as well as in long-axis orientations (slice thickness 8 mm, gap 2 mm, echo time 1.5 ms, repetition time 3.0 ms, flip angle 50°). For LGE imaging, an intravenous bolus of 0.2 mmol/kg gadolinium (Gadovist®, Bayer Inc., Toronto, Ontario, Canada) was administered. High-resolution (2 mm isotropic) iNav-3D LGE and 2D LGE using T1-weighted RF-spoiled gradient-echo inversion recovery sequences were performed in random order 10 min following contrast administration. The image-navigator approach has been previously implemented for coronary CMR angiography [19], and was acquired using the ramp-up profiles of the 3D LGE sequence, as illustrated in Fig. 1. The ramp-up scheme consisted of linearly increasing amplitude of 10 RF pulses prior to the 3D LGE sequence. Phase encoding gradients were added to the ramp-up profiles to enable iNAV acquisition, using a high-low profile order. A high-low profile order was used to obtain higher signal for the central k-space line of the navigator, which was acquired with the highest flip angle, as well as closest possible temporal distance to the 3D LGE acquisition. As the ramp-up profiles were used to generate the iNAVs, size, location and orientation of the field-of-view for the iNAVs and 3D LGE were identical. To maximize sensitivity to detect respiratory motion for the iNAVs, the field-of-view was oriented in the coronal plane covering the whole heart, with readout in foot-head direction (2 mm resolution) and phase encoding in left-right direction (9 mm resolution). In the slice-encoding direction the iNAVs were projections of the field-of-view resulting in a slice thickness of approximately 100–120 mm. iNAV motion compensation was implemented in real-time on the scanner and has been previously described [20]. In brief, the location of the shim geometry over covering the heart was used to define a region-of-interest in the first iNAV, which was used as reference iNAV. The reference was registered to every subsequent iNAV using 2D normalized cross-correlation, yielding translational motion in foot-head and left-right direction. The estimated motion was used to perform translational correction of the 3D LGE raw data by applying a linear phase shift. Respiratory gating was implemented with constant respiratory efficiency using single end-expiratory threshold CRUISE algorithm [18]. This approach acquires twice as much data as needed to fill 3D LGE k-space (resulting in exactly 50% gating efficiency) and only the half acquired at the most end-expiratory was used to reconstruct the gated image. Data acquisition of the iNAV-3D LGE was ECG-triggered to the mid-diastolic rest period of every cardiac cycle. The 2D LGE was acquired with multiple slices, covering the entire left ventricle in short axis orientation, in addition to single slices in two-, three- and four-chamber views. The 2D LGE consisted of a phase sensitive inversion recovery (PSIR) protocol with inversion pulses every other RR interval. Separate Look-Locker scans were performed prior to iNAV-3D and 2D LGE, to find the optimal inversion times to null healthy myocardium for one and two RR-interval inversion pulse frequencies, respectively. The scan times were recorded for both iNAV-3D and 2D LGE. For the 2D LGE, the scan time was recorded as the time between the acquisition of the first and last slice, including pauses between breath-holds to capture the true scan time of all 2D slices.

Table 1 describes the scan parameters for each method.

**Table 1:** Scan parameters for iNAV-3D and conventional 2D late gadolinium enhancement (LGE) methods. iNAV-3D: Image-Navigated High-Resolution 3-Dimensional, PSIR: phase sensitive inversion recovery

Parameter	iNAV-3D LGE	2D LGE
Inversion pulse	Every heart beat	Every 2nd heart beat
PSIR	No	Yes
Repetition time (ms)	4.8 ms	5 ms
Echo time (ms)	1.5 ms	1.8 ms
Flip angle	25	25
Parallel imaging factor	2	1.5
Field of view (mm^3^)	320 × 320 × 120 mm	320 × 320 × 80 mm
Acquired resolution (mm)	2.0 × 2.0 × 2.0 mm^3^	1.25 × 1.25 × 10 mm^3^
Reconstructed resolution (mm)	1.0 × 1.0 × 1.0 mm^3^	0.62 × 0.62 × 10 mm^3^
Nominal scan duration	1 min 58 s	15 s/ breath hold^a^

^a^: net duration of acquisition: 1 min 35 s, total scan duration including breath holds: 9 min 57 s

### CMR post processing

Post processing was performed with dedicated software (Circle Cardiovascular Imaging 4.2, Circle Cardiovascular Imaging, Calgary, Canada) by two independent cardiologists specializing in CMR imaging (7-years experience in CMR/ SCMR Level 3 accredited and 3-years experience in CMR/ SCMR Level 2 accredited, respectively), who were blinded to the results of prior measurements by the same reader, measurements by other readers and all clinical data, as well as the order in which the 2D and iNAV-3D LGE studies had been acquired. All 2D and 3D data were anonymised and presented in random order.

3D LGE image data were reformatted to short-axis planes to correspond with the 2D LGE slices and optimise comparisons. On both the 2D and iNAV-3D LGE images, endocardial and epicardial contours were automatically placed by the software and reviewed by the reader. After segmentation, diagnostic performance and image quality data analysis of 2D LGE and iNAV-3D images was performed. The acquired images were analyzed with regard to the agreement of the global and segmental detection, pattern and transmurality of LGE for each segment of a 16-segment model of the myocardium proposed by the American Heart Association excluding the apex [21].

**Fig. 2:**
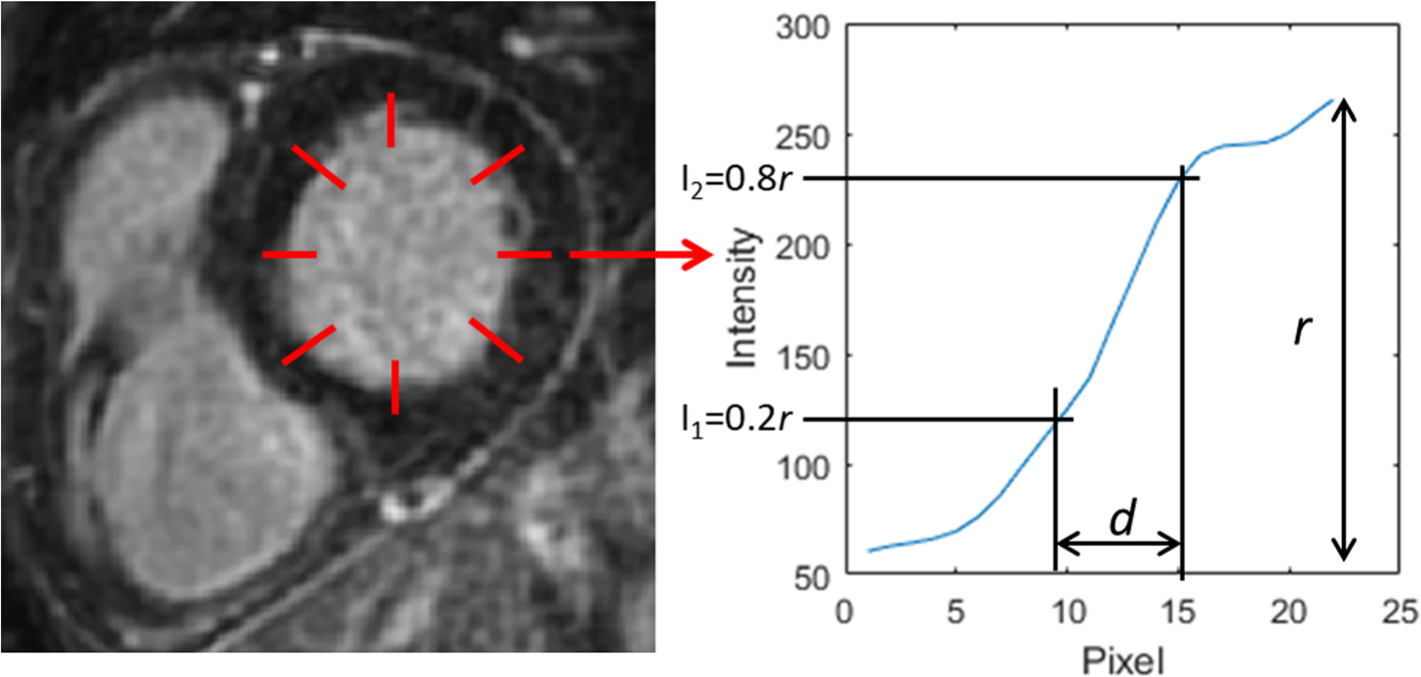
Example of endocardial border sharpness analysis in short axis view. Eight equidistant profiles are manually drawn perpendicular to the myocardium-blood interface, avoiding scar and myocardial trabeculations. For each profile, the image sharpness is defined as the pixel distance *d* between the 20% and 80% of the total intensity range *r*. The sharpness value was averaged across the 8 profiles for each volunteer and image type

Quantitative image sharpness was calculated for the 2D and 3D iNAV-LGE datasets. For each patient, a short-axis slice with the least amount of scar and myocardial trabeculations was selected for sharpness analysis. The corresponding slice in the iNAV-3D LGE was reformatted to the same reconstructed resolution. In each image, 8 profiles were manually selected along the endocardial border, at equidistant locations, as shown in Fig. 2. The sharpness was defined as the distance in pixels between 20% and 80% of the pixel intensity range of the profile, and a lower pixel distance indicate a sharper border. The sharpness from the 8 profiles was averaged for each dataset and patient. To ensure measurements were comparable between patients, all images were reformatted to the same in-plane resolution of 0.67 mm.

SNR was calculated for 2D LGE and iNAV-3D LGE for blood (SNR_blood_) and myocardium (SNR_myo_). The analysis was performed by drawing region of interests in the left ventricular blood pool and septum of the myocardium to obtain signal from the respective tissues. Noise was defined as the standard deviation of a region of air in the lungs (SD_air_), avoiding pulmonary vessels. The SNR values were calculated as the respective signal divided by SD_air_, multiplied by a factor of 0.655 to account for the Rayleigh distribution of the noise [22]. Furthermore, blood-myocardium contrast-to-noise (CNR_blood-myo_) was calculated using the same regions-of-interest as for the SNR calculations, subtracting SNR_myo_ from SNR_blood_, dividing by SD_air_ and multiplying by 0.655 to account for the noise distribution.

### Diagnostic quality scores

To obtain a head-to-head direct comparison between the two sequences only complete and diagnostic sequence sets were used for further analysis.

At first, visual perusal of the images was performed to determine whether infarction was present or absent. Positive finding in terms of a detectable scar in the LGE images was defined as a visible late enhancement. Global and segmental LGE detection were subsequently characterized in terms of LGE pattern (subendocardial/ ischemic, subepicardial, patchy, diffuse, involving right ventricular insertion points). In datasets with discrepancy between the two techniques, the results were compared against a panel standard. LGE transmurality was visually assessed by the reader as the segmental spatial extend of LGE within each segment and graded by using a five-point scale (0: 0%; 1: 1%–24%; 2: 25%–49%; 3, 50%–74%; 4, 75%–100%; 5, striae; 6- diffuse).

### Statistical analysis

Global agreement between the two acquisition techniques was assessed by means of McNemar test to account for patient pairing. Agreement between the two techniques in segmental scar detection was determined with a marginal homogeneity test. The difference in proportions for image quality, recoded as excellent/ non excellent, were evaluated by the McNemar test. The scan times were compared using paired t-test. The statistical analysis was performed using R 3.1.2. (R Project, Vienna, Austria). The statistical significance was assumed for *P* < 0.05.

## Results

### Baseline characteristics

A total of 23 patients were examined. Interpretable images were obtained in all 2D and in 22/23 iNAV-3D exams, resulting in a total of 22 complete sequence datasets and 352 segments. One iNAV-3D LGE dataset had to be discarded due to fast arrhythmia. The average acquisition time for iNAV-3D LGE was 3 min and 52 s with a standard deviation of 1 min and 39 s. Corresponding values for the stack of 2D LGE slices was 9 min and 57 s ± 2 min and 34 s. The difference in scan time was statistically significant (*P* < 0.001).

### Diagnostic quality

LGE was detected in 5 patients with ischemic pattern (23%), in 7 with non-ischemic pattern (32%), while it was absent in 10 cases (45%). There was excellent agreement between 2D and 3D data sets with regard to global LGE detection. There were no significant differences between the two techniques in segmental LGE detection and transmural extent (*P* = N.S.). The 95% confidence intervals and global tests showed no significant differences in the LGE pattern among all the considered combinations (*P* = N.S.). Similar observations were noted when the effect of the order of each sequence acquisition was considered.

**Fig. 3:**
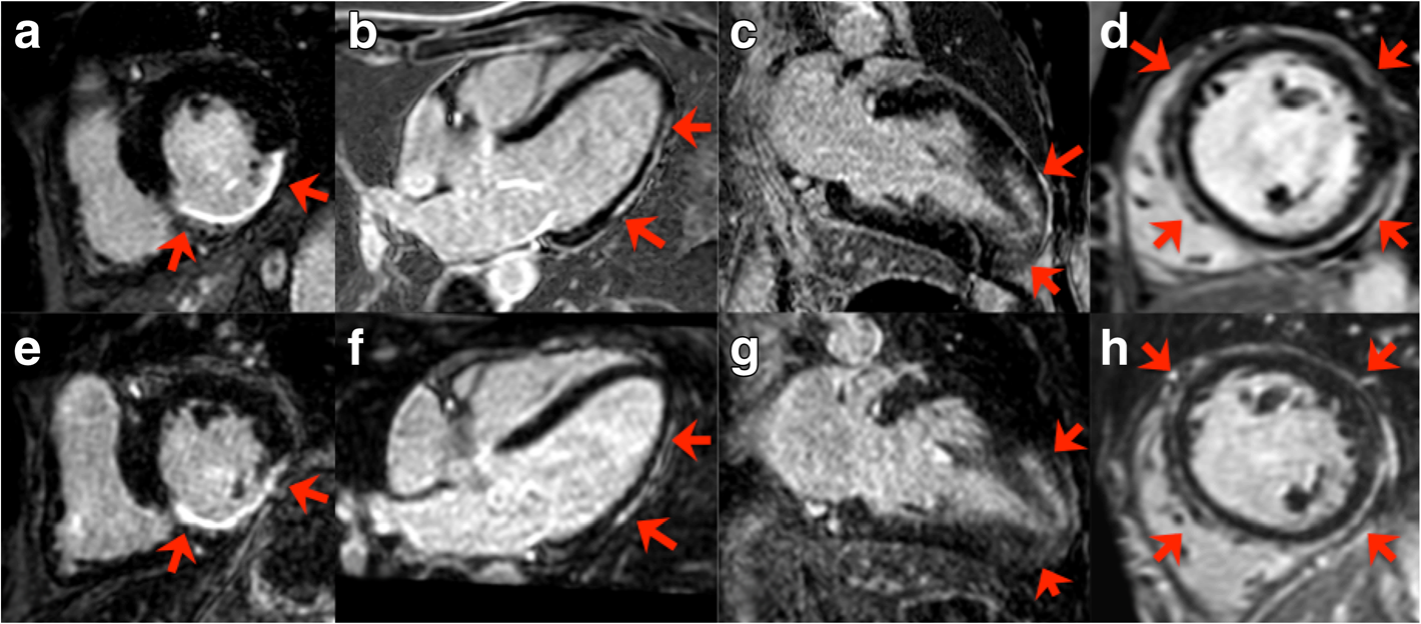
Selected matched images of 2D (upper row) and iNAV-3D (lower row) LGE in a patient with ischemic heart disease (**a** and **e**), myocarditis (**b** and **f**), hypertrophic cardiomyopathy (**c** and **g**) and dilated cardiomyopathy **(d** and **h**). Red arrows and star indicate the presence of LGE. Blurring due to residual respiratory motion is noticed in the latter three 3D–LGE images (**f-h**). Abbreviations as above

Table 2 summarizes the results of diagnostic quality assessment. Typical case examples in patients with LGE are provided in Fig. 3.

**Table 2:** Comparison of the main diagnostic quality scores for 2D and iNAV-3D LGE iNAV-3D LGE: Image-Navigated Late Gadolinium Enhancement, n: number, NS: not significant

Diagnostic performance							
Global LGE detection							
2D (*n* = 22)	55% (12)						
iNAV-3D (n = 22)	55% (12)						
Segmental LGE detection^a^ (*P* = 0.28)							
	0	1	2	3	4	5	
2D (*n* = 352)	75.9% (267)	10.2% (36)	3.7% (13)	7.7% (27)	2% (7)	0.6% (2)	
iNAV-3D (n = 352)	78.1% (275)	8.5% (30)	2% (7)	7.4% (26)	3.4% (12)	0.6% (2)	
LGE transmural extent^b^ (P = NS)							
	0	1	2	3	4	5	6
2D (n = 352)	268	6	7	10	13	14	34
iNAV-3D (n = 352)	275	3	3	5	19	30	17

^a^Segmental LGE detection: 0 = no LGE, 1 = ischaemic, 2 = patchy, 3 = subepicardial, 4 = mid wall, 5 = RV insertion points ^b^Transmural extension: 0, 1 = 1–25%, 2 = 26–50%, 3 = 51–75%, 4 = 76–100%, 5 = striae, 6 = diffuse

### Blood-myocardium sharpness

**Fig. 4:**
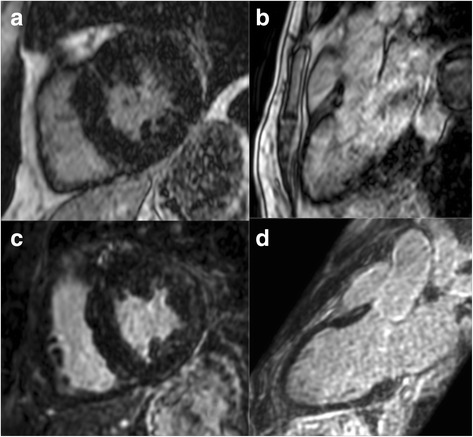
Selected matched images (**a-c, b-d**) of patients without LGE, showing improved image quality using iNAV-3D LGE (lower row) compared to 2D LGE (upper row). Abbreviations as above

The mean blood-myocardium sharpness ± standard deviation was 8.5 ± 3.6 for 2D LGE and 9.4 ± 3.0 for iNAV-3D LGE. No statistically significant differences were found between 2D LGE and iNAV 3D LGE for the blood-myocardium sharpness measurements (P = N.S.). Examples from two patients with improved image sharpness obtained using iNAV-3D LGE compared to 2D LGE are shown in Fig. 4.

### SNR and CNR

The SNR_blood_, SNR_myo_ and CNR_blood-myo_ for 2D LGE and iNAV-3D LGE are summarised in Table 3. Statistically significant differences were found for SNR_blood_ (*P* < 0.001) and CNR_blood-myo_ (*P* < 0.001), showing higher SNR_blood_ and CNR_blood-myo_ for 2D LGE, while SNR_myo_ was not statistically significant between 2D LGE and iNAV-3D LGE.

**Table 3:** Signal-to-noise (SNR) and contrast-to-noise (CNR) for blood and myocardium

	2D LGE	iNAV-3D LGE	*P*
SNR_blood_	26.1 ± 12.2	12.0 ± 3.8	0.001^a^
SNR_myo_	2.4 ± 1.1	2.1 ± 1.0	NS
CNR_blood-myo_	23.7 ± 11.1	9.9 ± 3.3	0.001^a^

^a^Denotes statistically significant differences. NS: not significant

## Discussion

In our study, we demonstrated the feasibility of using image-navigated 3D LGE imaging in a clinical setting for the detection of LGE. The diagnostic and image quality of the iNAV-3D LGE method were in very good agreement with conventional 2D LGE imaging.

Image-based navigation has previously been restricted to 3D coronary CMR angiography, but here we have adapted and extended the method to allow free-breathing high resolution 3D LGE as well. This study describes for the first time an advanced, image-based motion compensation technique deployed for 3D LGE in a clinical setting. The sequence was shown to shorten LGE scan time, while providing similar image quality based on a subjective assessment. With the image-navigated technique the respiratory position of the heart itself is monitored over time. The position of the heart at the beginning of each data segment is thus compared with a reference position, i.e. the position of the ventricular blood-pool at the very first data segment, and automatically corrected for respiratory motion prior to image reconstruction. The acquired volumetric data with isotropic resolution can be retrospectively reformatted and specific 2D images can be extracted in any plane orientation during post-processing.

Significant differences in SNR_blood_ and CNR_blood-myo_ were found between 2D LGE and iNAV-3D LGE, in favor of 2D LGE. This may be attributed to the smaller voxel size of iNAV-3D LGE (8 mm^3^) compared to 2D LGE (15.6 mm^3^). Furthermore, 2D LGE was acquired using inversion pulses every other RR-interval, leading to larger contrast between blood and myocardium compared to inversion repetitions every RR-interval that was used for iNAV-3D LGE. A limitation of the SNR and CNR analysis is that the noise estimation may be inaccurate due to the use of parallel imaging. Ideally, a noise scan should be acquired to accurately estimate the noise if parallel imaging is employed [23]. However, due to the additional scan time required to acquire the noise scan, this could not be included in this clinical study.

The image-navigated 3D cardiac imaging sequence is characterized by improved ease of use and a low operator interaction. This sequence yields high-resolution images and besides the detection of LGE, its quality supports its use to provide accurate estimates of total scar burden and to generate volumetric models of scar distribution. This is essential as the burden of myocardial scar in ischemic heart disease is an important predictor of functional recovery after revascularization [24] and particularly useful in the modern clinical trials where CMR infarct size is an indispensable surrogate endpoint.

The temporal resolution of iNAV-3D LGE can be individually adapted to the heart rate, resulting in a 3 to 5 min acquisition duration, which can be preset, compared to approximately 10 min for the stack of 2D LGE images. The significantly increased scan time using a stack of 2D LGE images is largely due to the need for patient recovery time between breath-holds, which substantially increases the overall scan time. Although the isotropic 3D scan requires reformatting to identify the conventional scan planes, this is done retrospectively and does not extend the scan time.

Furthermore, due to the non-isotropic resolution of 2D LGE, additional 2D slices are often required to visualize LGE, which is not adequately captured in standard short axis, two-, three- or four-chamber views, which further increase scan time. In contrast, iNAV-3D LGE is acquired with high isotropic resolution and with whole-heart coverage that enables retrospective reconstruction of the data in any view. The proposed free-breathing technique may potentially be of particular value as an alternative to conventional breath-held 2D LGE in patients with poor compliance.

A limitation of the proposed iNAV 3D LGE technique is that motion compensation is restricted to foot-head and left-right motion, ignoring any anterior-posterior motion of the heart. In 18/22 iNAV-3D LGE cases residual respiratory motion caused visible blurring **(**Fig. 3, f-h). As 2D LGE images were acquired during breath-hold, and therefore are motion free, in practice it may be technically extremely challenging to similarly completely eliminate respiratory motion in a generalizable motion compensation approach. The residual motion artifacts may explain the increased transmural extent of LGE measurements using iNAV-3D LGE, compared to 2D LGE, due to motion related blurring of the signal. Technical improvements including the development of 3D motion compensation algorithms [25–27] and 3D non-rigid motion correction [28, 29] may further improve image quality.

A further challenge of 3D LGE, which has not been addressed here, is the change in inversion time due to contrast washout during the scan [30]. Artifacts arising from sub-optimal inversion time from contrast washout may be exacerbated by the use of respiratory gating which prolongs the scan duration. Accelerated imaging may mitigate artifacts due to changes in inversion time and heart rate variability [31].

One dataset was discarded due to arrhythmia for iNAV 3D LGE and additional technical development is necessary to improve robustness to heart-rate variability and arrhythmia. This could include extension to PSIR LGE which is inherently more robust as the inversion pulse is performed every two heart-beats, leading to more stable signal even in the event of varying RR-intervals. Image-based navigation is particularly sensitive to arrhythmia, compared to conventional diaphragmatic navigation, as cardiac motion may be interpreted as respiratory motion. Additionally, signal and contrast fluctuations are more dramatic for inversion-recovery sequences in the event of arrhythmia, and this may also cause iNAV registration errors. Further work will focus on the implementation of arrhythmia rejection in conjunction with iNAV 3D LGE.

An advantage of the iNAV approach compared to conventional diaphragmatic navigator is that no navigator re-inversion pulse is required to restore the signal. This may be particularly useful for LGE of the pulmonary veins and left atrium where the navigator restore pulses cause signal enhancement mimicking LGE [32]. Nevertheless, due to the absence of navigator restore pulse the iNAV is susceptible to the same magnetization evolution as the 3D LGE, leading to a lower iNAV SNR. This may in turn impact the iNAV motion estimation precision. In this context, within the 10–25 min time window for LGE, performing iNAV-3D LGE earlier after contrast rather than later may be beneficial to improve signal from the blood pool, which could aid iNAV motion estimation. A limitation of this study is that no direct comparison between iNAV-3D LGE and diaphragmatic navigator was possible due to the time constraints of the CMR protocol. The diaphragmatic navigator approach, with a fixed, narrow gating window may result in low scan efficiency in the case of irregular breathing, leading to excessive scan times. In this study in patients referred for 2D LGE adding both iNAV-3D LGE and 3D LGE using conventional diaphragmatic navigator was not feasible. Further studies are required to directly compare the performance of iNAV and diaphragmatic navigator for 3D LGE.

There was no pathological reference standard for true infarct assessment. As our aim was to validate the feasibility of iNAV-3D method in the detection of late gadolinium enhancement in clinical practice comparison against a validated, conventional technique deemed sufficient. Patients with atrial fibrillation were excluded from the study, as conventional 2D LGE is known to be prone to arrhythmia-mediated artifacts and therefore the results would be significantly biased. The small sample size can hide some differences because of lack of statistical power. While the scope of the study was mainly to demonstrate feasibility of iNAV-3D LGE, further ongoing research projects recruiting larger number patients aim to more accurately determine diagnostic performance of this new technique.

## Conclusions

In this study, we have examined the feasibility of an image-navigated 3D LGE method in a clinical routine setting. The novel technique provides high isotropic spatial resolution LGE in a shorter scan time compared to conventional 2D LGE. The diagnostic quality of this imaging technique supports its use in clinical practice for accurate estimates of scar burden.

### Ethical approval

All procedures performed in studies involving human participants were in accordance with the ethical standards of the institutional and/or national research committee and with the 1964 Helsinki declaration and its later amendments or comparable ethical standards.

## Abbreviations

2D: Two-dimensional
3D: Three-dimensional imaging
CMR: Cardiovascular magnetic resonance
CNR: Contrast-to-noise ratio
ECG: Electrocardiogram
iNAV-3D: Image-navigated three-dimensional cardiac magnetic resonance imaging
LGE: Late gadolinium enhancement
PSIR: Phase sensitive inversion recovery
SNR: Signal-to-noise ratio
